# Racial differences in associations between baseline patterns of radiographic osteoarthritis and multiple definitions of progression of hip osteoarthritis: the Johnston County Osteoarthritis Project

**DOI:** 10.1186/s13075-015-0806-z

**Published:** 2015-12-18

**Authors:** Bridget Foley, Rebecca J. Cleveland, Jordan B. Renner, Joanne M. Jordan, Amanda E. Nelson

**Affiliations:** University of New England College of Osteopathic Medicine, Biddeford, ME USA; Thurston Arthritis Research Center, University of North Carolina at Chapel Hill, 3300 Doc J. Thurston Bldg, CB #7280, Chapel Hill, NC 27599-7280 USA; Department of Medicine, University of North Carolina at Chapel Hill School of Medicine, 3300 Doc J. Thurston Bldg, CB #7280, Chapel Hill, NC 27599-7280 USA; Department of Radiology, University of North Carolina at Chapel Hill School of Medicine, Chapel Hill, NC USA; Department of Epidemiology, Gillings School of Global Public Health, University of North Carolina at Chapel Hill, Chapel Hill, NC USA; Department of Orthopaedics, University of North Carolina at Chapel Hill School of Medicine, Chapel Hill, NC USA

**Keywords:** Hip, Osteoarthritis, Radiography, Disability

## Abstract

**Background:**

To identify baseline radiographic features that predict hip osteoarthritis (HOA) progression, and to explore differences in these associations by race.

**Methods:**

Radiographs from the community-based Johnston County OA Project were scored using Kellgren-Lawrence (KL) grade and for presence and location of joint space narrowing (JSN), osteophytes, and subchondral changes. Associations between these features and HOA progression (increase of at least 1 KL grade, interval hip replacement, range of motion [ROM, a reduction of ≥10° in internal rotation], or disability [increase of ≥0.2 in Health Assessment Questionnaire scores], or Any of these) were assessed using logistic regression, adjusting for age, gender, race, hip injury, BMI, education, smoking and follow-up time, accounting for multiple comparisons. Race interactions were assessed and analyses stratified as indicated.

**Results:**

The sample (n = 1,422) included 40 % men and 26 % African American (AA) participants, with mean age 61 years and BMI 29 kg/m^2^. The baseline frequency of radiographic hip OA (RHOA) between Caucasians and AAs was similar (23 %), although some radiographic features differed. AAs were more likely to have progression defined by ROM or disability or Any progression; Caucasians were more likely to have RHOA progression. JSN, subchondral sclerosis, and medial osteophytes were associated with increased RHOA progression overall; JSN was associated with disability progression only in AAs, while lateral osteophytes were associated with ROM progression only in Caucasians.

**Conclusions:**

AAs and Caucasians exhibited differences in the radiographic presentation and progression patterns of HOA, with AAs reporting progressive pain and disability, while Caucasians had more RHOA progression.

## Background

Osteoarthritis (OA) is a common, chronic condition that affects 11 % of the general adult population, and is the most common form of arthritis [1]. This percentage is expected to rise to 25 % by 2030, with 9.3 % of the population reporting activity limitation due to some type of arthritis [2]. OA is a disease process that encompasses the entire joint, most commonly involving the hips, knees, and hands, causing considerable pain and disability [3, 4]. Hip OA (HOA) in particular is associated with limitations in walking and climbing stairs and is the most common indication for total hip replacement surgery (THR) [5, 6]. Total hospital discharges for THR in the United States have been increasing in the last 20 years, with 286,324 discharges for THR in 1996, 369,372 in 2006 and 464,452 in 2011 (http://hcupnet.ahrq.gov).

There is a lack of standardization of the definitions of HOA, particularly for progression of this condition [7]. Progression in HOA has been measured in a variety of ways in previous studies, including (individually or in combination):

1. Decrease in radiographic joint space (either quantitative or qualitative) [8–19]
2. Increase in summary radiographic grade (Kellgren–Lawrence (KL) or others) [11–15, 20, 21]
3. Increase in total osteophyte score [11–15]
4. Receipt of THR [14–16, 18, 22, 23]
5. Worsening of self-reported pain or functioning [24]

Several baseline radiographic factors are associated with progression of HOA by various definitions. Joint space width ≤2.5 mm at study entry [8, 10, 18] is associated with progression of HOA defined by further joint space narrowing (JSN) [8, 10, 18] or THR [18]. Migration of the femoral head [8, 10], specifically superolateral migration [13, 23], has been associated with progression of HOA defined by progressive JSN [8, 10, 13], increase in summary grade [13], increase in total osteophyte score [13], or THR [8, 23]. Osteophytes have been associated with progression of HOA [13, 23] as defined by progressive JSN [13], increase in summary grade [13], increase in total osteophyte score [13], or THR [23]. Baseline hip pain [10, 13, 18, 23, 25] and increased disability scores [18] have also been associated with progression of HOA defined by progressive JSN [13, 18], increase in summary grade [13], increase in total osteophyte score [13] or THR [18, 23].

Although no significant difference in HOA prevalence was seen in the Johnston County OA Project (JoCo OA) [26, 27] or the First National Health and Nutrition Examination Survey (NHANES-I) [28], or for HOA progression in JoCo OA [20] between African Americans (AAs) and Caucasians, AAs have a consistently lower rate of THR utilization for treatment of HOA compared with Caucasians [29–31]. Racial differences in radiographic features of HOA have also been observed; specifically, mild axial JSN has been found more common in Caucasians than in AAs, while superior JSN, lateral osteophytes, and the presence of both acetabular and femoral osteophytes have been found more common in AAs [27]. Therefore, even though the overall frequency of progression may not differ significantly between AAs and Caucasians, disease progression may differ by race because of variations in baseline features of the disease. Our objective in this analysis was to identify baseline radiographic features that predict HOA progression using several different definitions, and to explore differences in these associations by race, using data from the JoCo OA.

## Methods

### Data collection

**Fig. 1:**
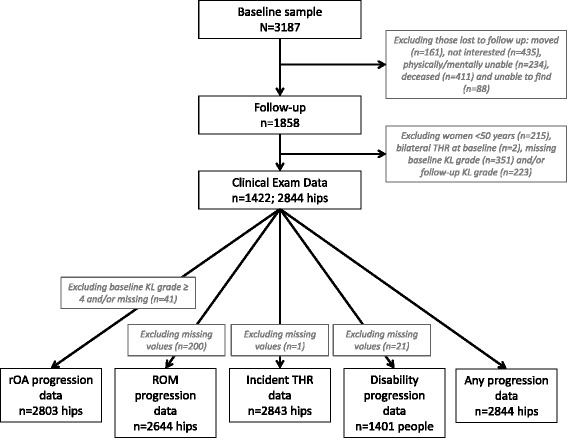
Strengthening the reporting of observational studies in epidemiology (STROBE) diagram of included participants and hips for each progression outcome. *THR* total hip replacement, *KL* Kellgren–Lawrence, *rOA* radiographic osteoarthritis, *ROM* range of motion

Data from the community-based JoCo OA cohort study in North Carolina were used. JoCo OA was designed to represent the civilian, non-institutionalized, AA and Caucasian population aged 45 years and older, living in one of six townships in Johnston County, NC for at least one year, and physically and mentally capable of completing the study protocol. Baseline data were collected between 1991 and 1997, with follow-up data collected between 1999 and 2003 [20, 26, 32]. From the original baseline sample of 3,187, 42 % were lost to follow up (Fig. 1); those that were lost to follow-up were more often AA, over 65 years of age, men, current smokers, and had less than a high school education. In addition, those who were lost to follow up reported more hip injuries at baseline, more depressive symptoms, and higher heath assessment questionnaire (HAQ) scores. There was no difference between those who remained in the study and those lost to follow up for hip OA, hip symptoms, THR, or obesity status. The sampling and methods of JoCo OA have been previously described [32]; the study has been continuously approved by the Institutional Review Boards of the University of North Carolina and the Centers for Disease Control and Prevention, and all participants provided detailed informed consent for the parent project, including use of data in other ancillary studies.

### Clinical features

Body mass index (BMI) was calculated in kg/m^2^ from height (cm) and weight (kg) measured during the physical examination. Age, gender, and race were self-reported. Educational attainment was included as an indicator of socioeconomic status. Symptoms were assessed separately for the right and left hips using the following question, administered by trained interviewers: “On most days, do you have pain, aching, or stiffness in your (right, left) hip?” [26, 32]; if answered affirmatively, participants rated these symptoms as mild, moderate, or severe. The Center for Epidemiologic Studies Depression Scale (CES-D) score was used as a measure of depressive symptoms [33]. Internal rotation in degrees was assessed for each hip using a goniometer. With the hip and knee flexed to 90°, the participant’s foot was rotated outward, and the angle was measured and recorded to the nearest degree by trained examiners. Pain on internal rotation was also assessed and recorded as mild (“patient states that it is painful”), moderate (“patient winces”), or severe (“patient withdraws”).

### Radiographic features

**Fig. 2:**
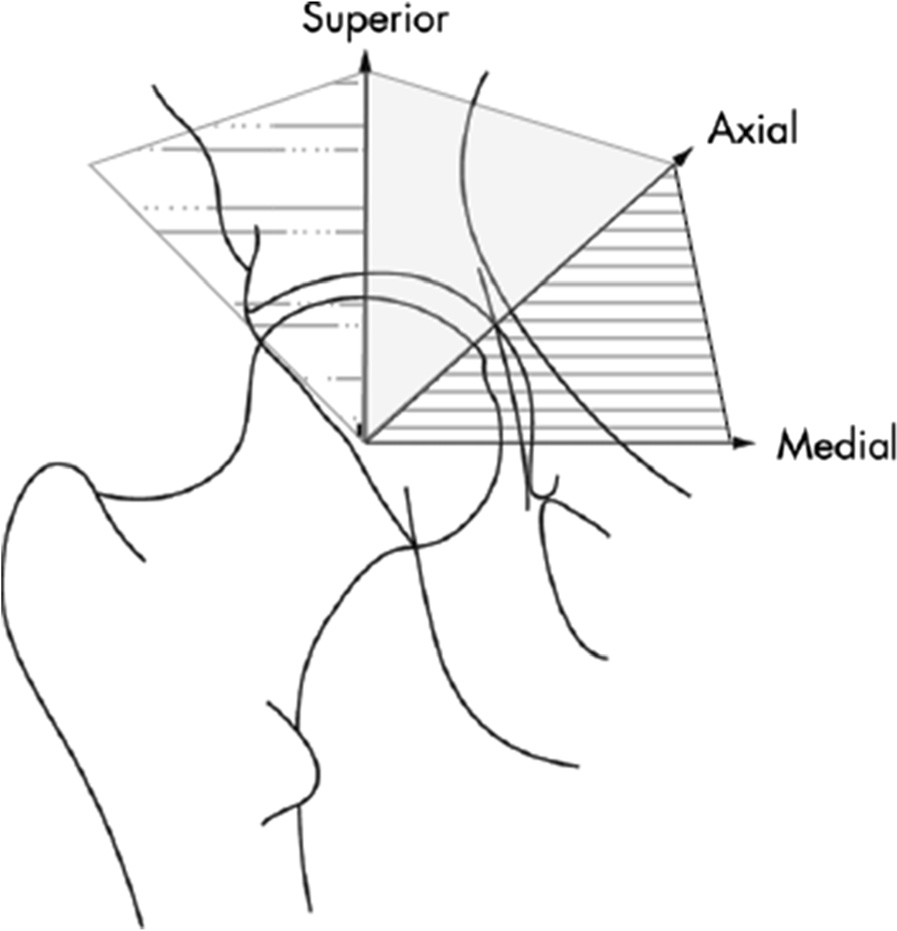
Depiction of superior, axial, and medial definitions of joint space narrowing (JSN). The region shaded with *dots* and *dashes* is defined as superior JSN, the *solid gray* as axial JSN, and the *horizontal lines* as medial JSN. Figure adapted from Lanyon, et al. Ann Rheum Dis 2004;63:259-63 [36]

Supine anteroposterior pelvic films, with the participant’s feet in 15° of internal rotation, were taken of all men, and of women ≥50 years of age. Baseline and follow-up hip radiographs were read as a pair by a single musculoskeletal radiologist (JBR), without knowledge of time point or participant clinical status. The KL radiographic atlas was used to assign overall hip radiographic grades [34]. Inter-rater and intra-rater reliability for KL grades for this reader were high (κ = 0.859 and 0.886, respectively), as previously described [35]. Radiographs scored as KL grade 0 (no HOA) showed no radiographic features of OA; KL grade 1 (questionable HOA) showed a small osteophyte of doubtful significance. KL grade 2 radiographs (mild HOA) showed an osteophyte, but no JSN. Radiographs showing moderate JSN were given a KL grade 3 (moderate HOA), and radiographs that included subchondral bone sclerosis and severe JSN were assigned a KL grade 4 (severe HOA) [34]. rHOA was defined as a KL grade ≥2. Four individual features of HOA were also assessed: presence of 1) JSN (superior, axial, medial or any combination of these,as defined by Lanyon et al. [36], Fig. 2); 2) subchondral cysts; 3) subchondral sclerosis; and 4) osteophytes (medial and lateral, either acetabular or femoral, or both acetabular and femoral), graded according to the Burnett atlas [37]. Reliability for identification of JSN and osteophytes was assessed separately in 60 individuals, with percentage agreement of 92 % and 95 %, and intra-rater kappa scores of 0.82 (95 % CI 0.67, 0.97) and 0.64 (0.27, 1.00), respectively. We were unable to analyze the severity of JSN and osteophytes due to extremely small numbers of participants with severe disease.

### Disability

Self-reported functional status was assessed with the Stanford HAQ disability index [38]. Participants scored 20 activities in 8 domains from 0 (no difficulty) to 3 (unable to do), with those activities requiring assistance to complete designated as a score of 2. The total HAQ score was calculated by using the highest score from each domain, then averaging the 8 domains [38].

### Progression

Progression was defined in four separate ways:

1. Radiographic (rHOA) progression: an increase in KL grade ≥1 (regardless of baseline KL)
2. Range of motion (ROM) progression: a reduction in internal rotation of the hip of ≥10° on standardized exam
3. THR progression: receipt of THR at follow up
4. Disability progression: an increase of 0.2 or more in HAQ score [38]

Or, given the variety of hip OA progression definitions in the literature, as any of the above (“Any progression”). We also considered increase in severity of hip symptoms, and an increase in pain severity with internal rotation as potential definitions of progression. We used an increase of at least one KL grade to define rHOA progression without requirement for a minimum baseline KL grade in order to avoid conditioning on an intermediate [39], allowing inclusion of all hips regardless of baseline KL grade and accounting for worsening of any magnitude. A novel definition of ROM progression was defined as a loss of at least 10° of internal rotation, based in part on clinical experience and known associations between internal rotation mobility and HOA [40]. In addition, Hando et al. described a 10° improvement in internal rotation following an 8-week physical therapy program for HOA that was also associated with a greater than minimal clinically important difference in Harris hip score [41], suggesting the clinical importance of this amount of change. This measure has been shown to be reliable [42], and was reliable at a later time point in the JoCo OA, with difference scores between two examiners <1° (in 40 random participants, difference scores were 0.21° (95 % CI –0.7, 1.13) on the left and –0.94° (95 % CI –1.64, 0.24) on the right; percent agreement for pain on internal rotation was also high: left 0.76 (95 % CI 0.63, 0.90), and right 0.79 (95 % CI 0.66, 0.92). THR is a widely accepted hard outcome in HOA progression, and disability progression was defined using an accepted cutoff for clinically meaningful worsening in HAQ score [38].

### Statistical analysis

Descriptive statistics were calculated to characterize the population for all variables of interest, including baseline demographics, baseline hip radiographic features, and HOA progression measures. Continuous variables were described using means with standard deviation (SD); categorical data were described using percentages.

Prevalent HOA measures were evaluated based on status at baseline, and progression was evaluated as the change from baseline to follow up. For the outcomes of pain increase and KL increase, individuals who had a prevalent highest level of pain (severe), or KL grade (KL = 4), respectively, at baseline were excluded because they were not eligible for progression of these measures. Analyses also excluded individuals who had both hips replaced at baseline, hips with THR at baseline, and hips missing baseline or follow up KL grades. Women under the age of 50 were also excluded because to avoid pelvic radiation they did not undergo hip radiography (Fig. 1).

With the exception of disability progression, all OA progression measures used were specific to each hip, and all analyses were based on the individual hip as the unit of analysis. We used generalized estimating equations (GEE) to address the potential correlation between right and left hip measurements within one person. We used logistic regression analysis to estimate odds ratios (OR) and 95 % CI for the strength of association between dichotomous baseline hip radiographic features and dichotomous OA progression. All models were adjusted for age, gender, race, prior hip injury, BMI (categorical data were used, although use of continuous BMI data did not affect the results), education, smoking and follow-up time. Analyses of rHOA progression were additionally adjusted for baseline KL grade, while analyses of disability progression were additionally adjusted for baseline CES-D scores. Bonferroni-adjusted alpha was set at *p* <0.013 for JSN predictors and *p* <0.006 for osteophyte predictors. Additional analyses were carried out to assess for interaction by race, and where significant interactions were identified (*p* ≤0.1), stratified analyses were performed. All statistical analyses were completed using SAS version 9.2 (SAS Institute Inc., Cary, NC, USA).

## Results

### Baseline characteristics

Baseline and follow-up data were available for 1,422 participants (Table 1, Fig. 1). The average age of the study group was 61.4 ± 9 years; 40 % were men and 26 % were AA. The majority of study participants were overweight or obese. One third of participants had not completed high school. The average time to follow up was 6.0 ± 1.4 years. Eight percent of participants met the criteria for at least mild depression, defined as a CES-D score >16 [33]. Mean HAQ score at baseline was 0.3 ± 0.5. AAs were more likely to be heavier, female, report less education beyond high school, currently smoke, and to have had longer times to first follow up.

**Table 1:** Demographic and clinical characteristics of baseline study participants

	All participants	Caucasian	AA	*P* value
Baseline demographic characteristics	n = 1422	n = 1054	n = 368	
Age, mean (SD), years	61.4 (9.0)	61.4 (8.8)	61.5 (9.4)	0.6967
BMI, mean (SD), kg/m^2^	28.9 (5.4)	28.3 (4.9)	30.6 (6.2)	<0.0001
<25 %	23.1	26.3	14.0	<0.0001
25 – <30 %	41.3	41.6	40.7	
30+ %	35.5	32.2	45.3	
Gender, % women	60.0	56.8	69.0	<0.0001
Educational attainment < HS, %	33.0	28.3	46.3	<0.0001
Smoking				
Never %	51.2	49.4	56.3	<0.0001
Past %	32.0	35.2	22.8	
Current %	16.8	15.4	20.9	
Hip injury, %	2.4	2.3	2.6	0.5723
CES-D <16, %	8.0	7.4	9.9	0.0315
HAQ, mean (SD), score	0.3 (0.5)	0.3 (0.5)	0.3 (0.5)	0.1992
Follow-up time, mean (SD), years	6.0 (1.4)	5.8 (1.3)	6.5 (1.4)	<0.0001

*AA* African American, *BMI* body mass index, *HS* high school, *CES-D* Center for Epidemiologic Studies depression scale, *HAQ* health assessment questionnaire

As shown in Table 2, at baseline, 23 % of participants had definite rHOA ranging from mild to severe (KL grade 2–4). At baseline, axial JSN (Fig. 2) was most frequent, while medial JSN was least frequent. Subchondral sclerosis was seen more frequently than subchondral cysts. Osteophytes were observed on the femoral and acetabular sides of the joint, both laterally and medially; lateral acetabular osteophytes were most common (Table 2).

**Table 2:** Hip features of study participants at baseline

Hip radiographic feature at baseline	All participants	Caucasian	AA	*P* ^a^
	n = 1422	n = 1054	n = 368	
KL grade				
0: No hip OA %	15.3	17.3	9.5	<0.0001^b^
1: Questionable hip OA %	61.6	59.3	68.5	
2: Mild hip OA %	21.6	22.2	19.7	
3: Moderate hip OA %	1.3	1.1	1.9	
4: Severe hip OA %	0.2	0.1	0.4	
JSN				
Axial				
None %	77.7	75.9	82.9	<0.0001
Mild %	21.6	23.7	15.7	
Moderate/severe %	0.7	0.4	1.4	
Superior				
None %	91.6	92.7	88.5	0.0011
Mild %	7.8	6.9	10.4	
Moderate/severe %	0.6	0.4	1.1	
Medial				
None %	98.2	98.2	98.1	0.8599^b^
Mild %	1.8	1.7	1.9	
Moderate/severe %	0.1	0.1	0	
Subchondral				
Cysts %	4.1	3.9	4.5	0.5193
Sclerosis %	14.1	14.5	13.1	0.3337
Medial osteophytes				
Severity				
None %	92.1	92.6	90.8	0.2221
Mild %	6.8	6.3	8.2	
Moderate/severe %	1.1	1.1	1.0	
Location				
None %	92.2	92.6	91.0	0.0353
Acetabular only %	2.9	2.4	4.4	
Femoral only %	3.5	3.7	3.0	
Both %	1.4	1.3	1.6	
Lateral osteophytes				
Severity				
None %	42.8	46.0	33.9	<0.0001
Mild %	48.6	46.5	54.6	
Moderate/severe %	8.5	7.5	11.4	
Location				
None %	42.8	46.0	33.7	<0.0001
Acetabular only %	40.8	38.2	48.1	
Femoral only %	4.7	5.1	3.6	
Both %	11.7	10.7	14.7	

^a^Chi-square or Fisher’s Exact p-value comparing Caucasians to African Americans (*AAs*). ^b^Fisher’s exact test *p* value. *KL* Kellgren–Lawrence, *OA* osteoarthritis, J*SN* joint space narrowing

#### Racial differences at baseline

Racial differences were noted at baseline (Table 2). While the prevalence of definite rHOA was similar between AA and Caucasian participants, AAs were more likely to have a KL grade of 1, and significantly less likely to have a KL grade of 0 compared with Caucasians. Axial JSN was significantly more common in Caucasian compared with AA participants, while superior JSN was more common in AAs. No significant racial difference was seen for medial JSN, subchondral bone changes, or medial osteophytes, but AAs were more likely to have lateral osteophytes (Table 2).

### Follow-up characteristics

**Fig. 3:**
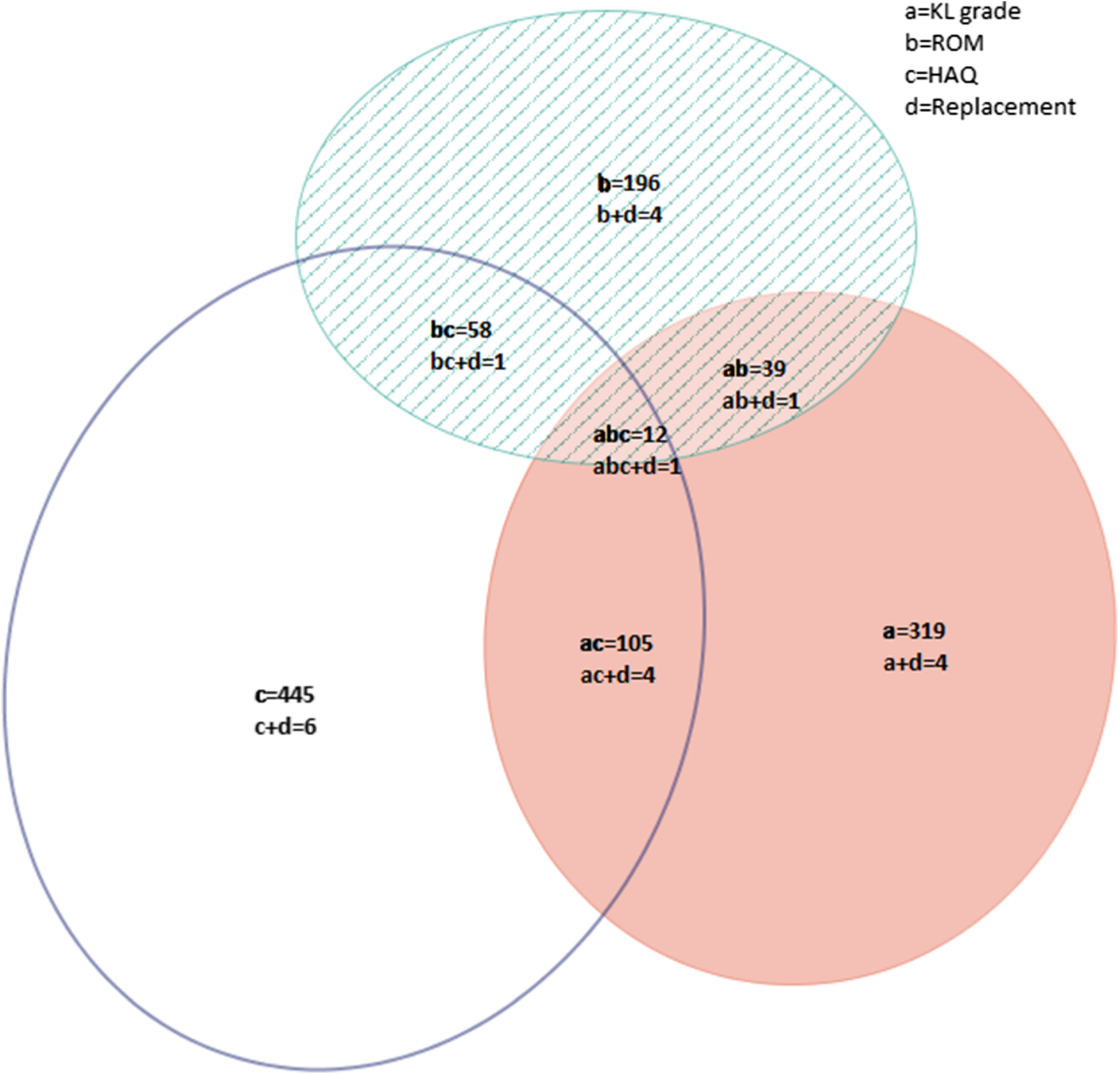
Overlap between different definitions of hip osteoarthritis progression. *KL* Kellgren–Lawrence, *ROM* range of motion, *HAQ* health assessment questionnaire

At follow up, 15 % of hips had undergone rHOA progression, including 16 % of hips in Caucasians and 11 % of hips in AAs (*p* = 0.0007, Table 3). Progression occurred in 15 % of hips according to the ROM definition; 14 % of hips in Caucasians, and 19 % of hips in AAs (*p* = 0.0012). Twelve individuals had undergone THR with no significant difference by race (*p* = 0.742). Compared with Caucasians, AAs were more likely to have a higher frequency of any progression (55 % vs. 48 %, respectively), and to have an increase of at least 0.2 points in HAQ score (40 % vs. 29 %, respectively, *p* <0.0001). Overlap between these definitions is depicted in the Venn diagram in Fig. 3.

**Table 3:** Hip OA progression by individual definitions

OA progression indicators	All participants	Caucasian	AA	*P* ^a^
	n (%)	n (%)	n (%)	
Radiographic OA progression^b^ (n = 2,803 hips)	411 (14.7)	332 (16.0)	79 (10.8)	0.0007
Range of motion progression^c^ (n = 2,644 hips)	404 (15.3)	279 (14.0)	125 (19.3)	0.0012
Incident hip replacement at T1 (n = 2,843 hips)	12 (0.4)	10 (0.5)	2 (0.3)	0.742^d^
Disability progression^e^ (n = 1,401 participants)	445 (31.8)	302 (29.0)	143 (39.7)	<0.0001
Any progression (n = 2844 hips)	1,406 (49.4)	1,003 (47.6)	403 (54.8)	0.0008

^a^Chi-square or Fisher’s exact test *p* value comparing Caucasians to African Americans (*AA*). ^b^Kellgren–Lawrence grade increased by at least 1 from time point 0 (T0) to time point 1 (*T1*). ^c^Internal rotation decrease ≥10° in one or both hips. ^d^Fisher’s exact test *p* value. ^e^Health assessment questionnaire score increase > =0.2. *OA osteoarthritis*

### Associations with progression

#### Joint space narrowing

Those with JSN at any site (axial, medial or superior, Table 4) were almost three times as likely to have rHOA progression, 30 % more likely to have ROM progression, and thirteen times as likely to have received a THR at follow up. JSN was not associated with disability progression, and was modestly associated with any progression. After correction for multiple comparisons, the association between JSN at any site and rHOA progression (*p* <0.0001) was still statistically significant, but association with ROM progression and THR was not. JSN at any site was associated with disability progression in AAs (adjusted OR (Aor) = 1.71, 95 % CI (1.06, 2.74), interaction *p* value = 0.037) but not in Caucasians (aOR 0.92, 95 % CI (0.68, 1.23)); no interactions were seen for other progression outcomes and JSN.

**Table 4:** Adjusted odds ratios (aORs) for associations between patterns of JSN and progression

	rOA progression	ROM progression	THR	Disability progression	Any progression
JSN	(n = 411 hips)	(n = 404 hips)	(n = 12 hips)	(n = 445 participants)	(n = 1406 participants)
Any location	2.62 (1.95, 3.51)^a^	1.31 (1.05, 1.63)	12.9 (1.63, 102)	1.06 (0.83, 1.36^b^	1.20 (1.03, 1.40)
Axial	2.05 (1.44, 2.91)^a^	1.30 (1.00, 1.68)	19.9 (2.50, 159)	0.86 (0.66, 1.12)	1.11 (0.92, 1.33)
Superior	2.75 (1.79, 4.24)^a^	1.11 (0.81, 1.53)	1.78 (0.45, 6.99)	1.50 (1.07, 2.09)	1.42 (1.11, 1.82)
Medial	4.51 (1.47, 13.8)	0.68 (0.33, 1.40)	39.2 (7.91, 194)^a^	1.40 (0.69, 2.88)	1.56 (0.76, 3.21)

All models were adjusted for age, gender, race, prior hip injury, body mass index, education, smoking and follow-up time; the progression of radiographic osteoarthritis (rOA) outcome was additionally adjusted for baseline Kellgren–Lawrence grade, while the disability outcome was also adjusted for the Center for Epidemiologic Studies Depression Scale. ^a^Statistically significant after Bonferroni adjustment of *p* values. ^b^Significant interaction (*p* = 0.037) by race: for African Americans the aOR = 1.71, 95 % CI (1.06, 2.74), for Caucasians the aOR = 0.92, 95 % CI (0.68, 1.23). *JSN* joint space narrowing, *ROM* range of motion, *THR* total hip replacement

By location, axial JSN was positively associated with rHOA and ROM progression in adjusted analyses. Axial JSN was also associated with THR, but with a very wide CI. There were no associations between axial JSN and disability or any progression. Using Bonferroni-adjusted *p* values for multiple comparisons, the association between axial JSN and RHOA progression (*p* = 0.0001) was still statistically significant, but those with ROM progression and THR were not (Table 4). Superior JSN was associated with rHOA progression, disability progression, and any progression. There were no statistically significant associations between superior JSN and ROM progression or THR. After adjustment for multiple comparisons, the associations found between superior JSN and rHOA progression (*p* <0.0001) was still statistically significant, but the association with disability progression and any progression were not. Medial JSN, though infrequent in the sample, was the most strongly associated with rHOA progression and with THR. There was no association between medial JSN and ROM progression, disability progression, or any progression. After correction for multiple comparisons, only the association between THR and medial JSN (*p* <0.0001) was still statistically significant.

#### Subchondral bone changes

Subchondral cysts were associated only with rHOA progression (aOR = 1.83, 95 % CI (1.01, 3.31)), but this association was no longer significant after adjustment for multiple comparisons. Subchondral sclerosis was associated with rHOA progression (aOR = 2.09, 95 % CI (1.4, -3.03)) and with THR (aOR = 5.95, 95 % CI (1.78, 19.9)), but no associations were seen with ROM, disability, or any progression. Using Bonferroni-adjusted *p* values for multiple comparisons, the association between subchondral sclerosis with rHOA progression and THR was still statistically significant (*p* = 0.0001 and *p* = 0.0038, respectively). There was a stronger association between subchondral sclerosis and rHOA progression in AAs (aOR 3.64, 95 % CI (1.61, 8.21)) than in Caucasians (aOR = 1.81, 95 % CI (1.19, 2.75), interaction *p* value = 0.069); no interactions were seen for other progression outcomes and subchondral changes.

#### Osteophytes

Those with osteophytes at any site (medial, lateral, acetabular, femoral) had 50 % higher odds of ROM progression, and 16 % lower odds of any progression (Table 5). Those with any medial osteophytes were three times as likely to have rHOA progression, seventeen times as likely to have THR, and 40 % more likely to have any progression. Medial acetabular osteophytes were associated with rHOA progression, ROM progression, and THR but not with disability or any progression. Medial femoral osteophytes were associated only with rHOA progression and THR. The combination of both medial acetabular and femoral osteophytes was associated with THR but was protective against ROM progression. Using Bonferroni-adjusted *p* values for multiple comparisons, the associations between rHOA progression and any medial osteophytes (*p* <0.0001), and medial femoral osteophytes (*p* = 0.0005) remained statistically significant. Additionally, the associations of any medial osteophytes or any femoral osteophytes, or both with THR (*p* <0.0001) remained statistically significant after correction for multiple comparisons.

**Table 5:** Adjusted odds ratios (aORs) for associations between patterns of osteophytes and progression

	rOA progression	ROM progression	THR	Disability progression	Any progression
Osteophytes	(n = 411 hips)	(n = 404 hips)	(n = 12 hips)	(n = 445 participants)	(n = 1406 participants)
Any site	0.99 (0.77, 1.29)	1.51 (1.19, 1.92)*	1.87 (0.40, 8.83)	1.05 (0.81, 1.36)	0.84 (0.72, 0.97)
Medial					
Any site	2.99 (1.93, 4.64)*	1.27 (0.89, 1.81)	17.4 (4.80, 63.1)*	1.28 (0.89, 1.84)	1.39 (1.05, 1.83)
Any acetabular	2.80 (1.34, 5.83)	1.58 (1.04, 2.42)	5.33 (1.01, 28.1)	1.39 (0.78, 2.50)	1.40 (0.98, 2.02)
Any femoral	2.63 (1.52, 4.54)*	0.72 (0.46, 1.12)	13.5 (3.23, 56.5)*	1.66 (0.96, 2.85)	1.19 (0.85, 1.68)
Both acetabular and femoral	2.55 (0.52, 12.6)	0.41 (0.20, 0.84)	30.3 (4.25, 216)*	1.40 (0.51, 3.85)	0.97 (0.49, 1.95)
Lateral					
Any site	0.95 (0.73, 1.23)	1.40 (1.11, 1.77)^†a^	1.15 (0.30, 4.44)	1.04 (0.80, 1.34)	0.82 (0.71, 0.95)
Any acetabular	0.94 (0.71, 1.24)	1.45 (1.15, 1.84)*^†b^	1.67 (0.48, 5.78)	0.99 (0.78, 1.26)	0.84 (0.71, 1.00)
Any femoral	1.43 (1.03, 1.97)	0.95 (0.72, 1.26)^†c^	4.09 (1.28, 13.1)	1.18 (0.88, 1.58)	1.11 (0.92, 1.33)^†d^
Both acetabular and femoral	1.65 (1.12, 2.41)	1.00 (0.71, 1.39)	4.08 (1.24, 13.4)	1.19 (0.85, 1.67)	1.21 (0.97, 1.50)^†e^

All models adjusted for age, gender, race, prior hip injury, body mass index, education, smoking and follow-up time; the radiographic osteoarthritis (*rOA*) outcome was additionally adjusted for baseline Kellgren–Lawrence grade, while the disability outcome was adjusted also for the Center for Epidemiologic Studies depression scale. *Statistically significant after Bonferroni *p* value adjustment. ^†^Significant interactions by race. ^†a^For AAs aOR = 0.99, 95 % CI (0.64, 1.53), for Caucasians aOR 1.65, 95 % CI (1.25, 2.17), interaction *p* = 0.038. ^†b^For AAs aOR = 1.10, 95 % CI (0.72, 1.68), for Caucasians aOR = 1.65, 95 % CI (1.25, 2.19), interaction *p* = 0.091. ^†c^For AAs aOR = 0.58, 95 % CI (0.34, 0.98), for Caucasians aOR = 1.16, 95 % CI (0.83, 1.61), interaction *p* = 0.015. ^†d^For AAs aOR = 0.83, 95 % CI (0.58, 1.19), for Caucasians aOR = 1.23, 95 % CI (0.99, 1.52), interaction *p* = 0.056. ^†e^For AAs aOR = 0.87, 95 % CI (0.59, 1.28), for Caucasians aOR = 1.33, 95 % CI (1.01, 1.76), interaction *p* = 0.067 *ROM* range of motion, *THR* total hip replacement

Those with any lateral osteophytes were 40 % more likely to have ROM progression, but were 18 % less likely to have any progression. The presence of lateral acetabular osteophytes was associated with ROM progression, while lateral femoral osteophytes were associated with rHOA progression and THR at follow up. The presence of lateral osteophytes on both the acetabular and femoral sides was associated with rHOA progression and THR but no other progression definitions. Using Bonferroni-adjusted *p* values for multiple comparisons, the association between the presence of lateral acetabular osteophytes and ROM progression was still statistically significant, but other associations were not.

The presence of lateral osteophytes was associated with ROM progression in Caucasians (aOR = 1.65, 95 % CI (1.25, 2.17), interaction *p* value = 0.038) but not in AAs (aOR 0.99, 95 % CI (0.64, 1.53)). Similar associations were seen for lateral acetabular osteophytes. Lateral femoral osteophytes were potentially protective against ROM progression in AAs, but not in Caucasians at follow up (AA aOR = 0.58, 95 % CI (0.34, 0.98), Caucasian aOR = 1.16, 95 % CI (0.83, 1.61), interaction *p* value = 0.015), and were associated with any progression in Caucasians only (AA aOR = 0.83, 95 % CI (0.58, 1.19), Caucasian aOR = 1.23, 95 % CI (0.99, 1.52), interaction *p* value = 0.056). The combination of acetabular and femoral osteophytes together was also associated with any progression only in Caucasians (AA aOR = 0.87, 95 % CI (0.59, 1.28), Caucasian aOR = 1.33, 95 % CI (1.01, 1.76), interaction *p* value = 0.067).

There were no associations between any of the radiographic features and either increased hip symptoms or increased pain on internal rotation (data not shown).

## Discussion

All investigated radiographic features were associated with at least one of the four HOA progression definitions; JSN, bony sclerosis, and osteophytes were all associated with multiple progression outcomes.

### Baseline racial differences

Our group previously identified racial differences in radiographic features using cross-sectional baseline JoCo OA data. Specifically, there was a higher frequency of mild axial JSN in Caucasians, superior JSN in AAs, and higher frequency of lateral osteophytes in AAs [27]. The current study confirms these findings in a sample restricted to 1,422 study participants who had paired radiographic readings for baseline and follow up.

### Association with progression: JSN

Several studies have identified positive associations between quantitative joint space width (JSW) at baseline and HOA progression at follow up defined as JSN or THR [8–10, 18, 43]. These studies did not differentiate between axial, superior, and medial narrowing. We identified differences in associations with progression outcomes based on patterns of JSN such that axial JSN was associated with rHOA and ROM progression, but superior JSN was associated with rHOA and disability progression. Because only a small number of hips were replaced, the observed associations between JSN and THR had very wide confidence intervals and are likely unstable; this notwithstanding, associations between axial and medial JSN and THR were very strong. Lane et al., using data from the Study of Osteoporotic Fractures (SOF), reported associations between superolateral JSN (comparable to superior JSN in our study) and THR or a decrease of at least 0.5 mm in minimum JSW; superomedial JSN (comparable to axial JSN) was associated with increased risk of THR but appeared to be protective against an increase in summary OA grade or increase in osteophyte score [13]. Differences in the two studies may reflect differences in follow-up time (8 years for SOF, 6 years for JoCo OA), population (Caucasian women in SOF versus AA and Caucasian men and women in the current study), grading scheme (Croft versus KL) and age (mean 72 years for SOF and 61 years for JoCo OA). Additionally, the SOF examined only the 745 women who had rHOA at baseline for progression, while in the current study we included all 1,422 participants with paired films, therefore likely capturing earlier stages of incident and progressive HOA.

### Associations with progression: osteophytes and subchondral changes

Previous studies have identified positive associations between osteophytes and progression (defined as JSN, THR, composite definitions, or increase in KL grade) [10, 13, 23]. We found positive associations between medial acetabular osteophytes and both rHOA and ROM progression. Lateral acetabular osteophytes, which may lead to pincer impingement [44, 45], were also associated with ROM progression. We found positive associations between lateral femoral osteophytes and rHOA progression and THR. Medial femoral osteophytes were also associated with rHOA progression. Association was reported between femoral osteophytes and all progression outcomes assessed in the SOF, while acetabular osteophytes were not statistically significantly associated with progression by any definition [13]. Osteophytes in that study, however, were not differentiated by medial or lateral location. Like Lane et al., we found associations between cysts and sclerosis and rHOA progression.

### Racial differences

In this follow up to our 2010 cross-sectional analysis [27], we were interested in determining the impact of racial differences in radiographic features at baseline in this population on the course of hip OA after approximately 6 years of follow up. Given the higher frequency of osteophytosis and superior JSN in AAs, which had previously been associated with increased THR utilization in another study [13], we hypothesized that AAs might have a higher risk of progression compared with Caucasians. In the current study, although AAs were again noted to have a similar prevalence of RHOA compared with Caucasians, AAs had less rHOA progression, but more frequent disability and ROM progression. Therefore, indications for THR in this group may be more related to alterations in physical function and disability rather than progressive radiographic change. There are complex and multifactorial issues surrounding disparities in THR utilization that also affect this issue and are well-reviewed in the literature [46–49].

This study had several limitations, including a small sample size for THR outcomes (<1 % had undergone THR at follow up) leading to wide confidence intervals for these estimates. We did not have continuously measured quantitative joint space width, but did have semiquantitative measures of JSN available. Our study also has many strengths. This large cohort included both AA and Caucasian men and women and was community-based. The data are from standardized questionnaires, physical examinations of clinically relevant outcomes with high reliability, and paired radiographs, including detailed radiographic features with high reliability from a single musculoskeletal radiologist. This study, in comparison to prior work, included a more in-depth analysis of the location of osteophytes and JSN in regards to HOA progression and assessed multiple definitions of progression including radiographic and clinical features.

## Conclusions

AAs and Caucasians exhibited differences in baseline hip radiographic features with implications for HOA progression. AAs reported increased pain and disability after 6 years of follow up, while Caucasians had more rHOA progression. Worsening of disability in association with baseline radiographic features in AAs is supportive of a potential unmet need for hip OA management in this population.

## Abbreviations

AA: African American
aOR: adjusted odds ratio
BMI: body mass index
CES-D: Center for Epidemiologic Studies depression scale
HAQ: health assessment questionnaire
HOA: hip osteoarthritis
JoCo OA: Johnston County Osteoarthritis Project
JSN: joint space narrowing
KL: Kellgren–Lawrence
OA: osteoarthritis
OR: odds ratio
rHOA: radiographic hip osteoarthritis
ROM: range of motion
SOF: Study of Osteoporotic Fractures
THR: total hip replacement
